# Supplementary material for: A whole-animal phenotypic drug screen identifies suppressors of atherogenic lipoproteins
Source: eLife. 2026 Aug 27;14:RP105314. doi: 10.7554/eLife.105314 (PMC13521566; doi:10.7554/eLife.105314)
Supplement: Supplementary file 2 [file elife-105314-code1.zip › JHDL_primary_result/Demecarium bromide/Demecarium bromide_JHDL_35_024_069_2020_01_31_12_41.pdf]

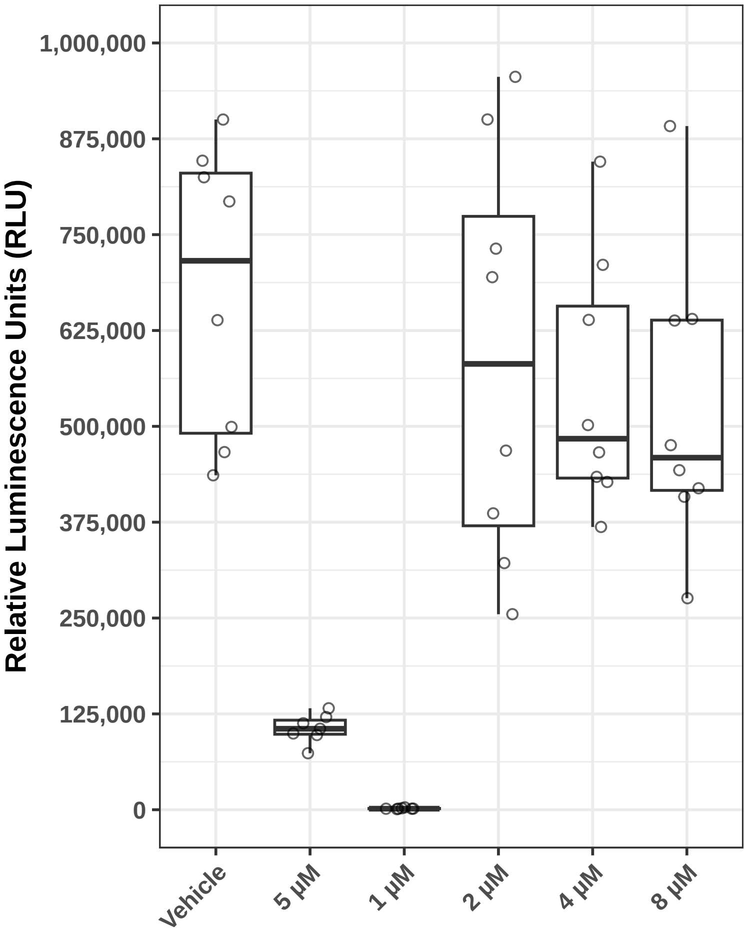
Relative Luminescence Units (RLU)
1,000,000
875,000
750,000
625,000
500,000
375,000
250,000
125,000
0
Vehicle
5 µM
1 µM
2 µM
4 µM
8 µM
